# *Carica papaya* MicroRNAs Are Responsive to *Papaya meleira virus* Infection

**DOI:** 10.1371/journal.pone.0103401

**Published:** 2014-07-29

**Authors:** Paolla M. V. Abreu, Clicia G. Gaspar, David S. Buss, José A. Ventura, Paulo C. G. Ferreira, Patricia M. B. Fernandes

**Affiliations:** 1 Núcleo de Biotecnologia, Universidade Federal do Espírito Santo, Vitória, Espírito Santo, Brazil; 2 Instituto de Bioquímica Médica Leopoldo de Meis, Universidade Federal do Rio de Janeiro, Rio de Janeiro, Brazil; 3 Instituto Capixaba de Pesquisa, Assistência Técnica e Extensão Rural, Vitória, Espírito Santo, Brazil; Universidade Federal do Rio Grande do Sul, Brazil

## Abstract

MicroRNAs are implicated in the response to biotic stresses. *Papaya meleira virus* (PMeV) is the causal agent of sticky disease, a commercially important pathology in papaya for which there are currently no resistant varieties. PMeV has a number of unusual features, such as residence in the laticifers of infected plants, and the response of the papaya to PMeV infection is not well understood. The protein levels of 20S proteasome subunits increase during PMeV infection, suggesting that proteolysis could be an important aspect of the plant defense response mechanism. To date, 10,598 plant microRNAs have been identified in the Plant miRNAs Database, but only two, miR162 and miR403, are from papaya. In this study, known plant microRNA sequences were used to search for potential microRNAs in the papaya genome. A total of 462 microRNAs, representing 72 microRNA families, were identified. The expression of 11 microRNAs, whose targets are involved in 20S and 26S proteasomal degradation and in other stress response pathways, was compared by real-time PCR in healthy and infected papaya leaf tissue. We found that the expression of miRNAs involved in proteasomal degradation increased in response to very low levels of PMeV titre and decreased as the viral titre increased. In contrast, miRNAs implicated in the plant response to biotic stress decreased their expression at very low level of PMeV and increased at high PMeV levels. Corroborating with this results, analysed target genes for this miRNAs had their expression modulated in a dependent manner. This study represents a comprehensive identification of conserved miRNAs inpapaya. The data presented here might help to complement the available molecular and genomic tools for the study of papaya. The differential expression of some miRNAs and identifying their target genes will be helpful for understanding the regulation and interaction of PMeV and papaya.

## Introduction

Noncoding RNAs comprise the majority of transcribed RNA, and they perform a wide range of functions in cellular and developmental processes. As a result, noncoding RNAs are also implicated in the development and pathophysiology of many diseases, and they represent potential targets for therapeutic intervention. Plant disease management strategies consist of two principles, prevention and therapy (treatment or cure). For both of these approaches, the use of disease-resistant plants is the ideal. The development of disease-resistant plants has been relatively successful when the proper knowledge of the plant-pathogen interaction system is available.

MicroRNAs (miRNAs) are one of a number of classes of endogenous, small (21–24 nucleotide), non-coding RNAs found in both animals and plants [1], [2]. In plants, these classes include miRNAs and small interfering RNAs (siRNAs), which are distinguished by their precursors. SiRNAs are derived from double stranded RNA (dsRNA), including viral RNA, and they destroy their parent molecules, a process termed “autosilencing” [3]. In contrast, miRNAs are endogenous to the plant, and they are produced by genes that are distinct from those that they regulate, by a process termed “heterosilencing” [3]. Precursors to plant miRNAs vary from approximately 70 to 180 nt in length [4], and they generally harbour a characteristic stem and loop structure, although branched structures have been found [4]. MiRNAs are processed by Dicer-Like 1 protein (DCL1) [5] to form active miRNAs, which are then methylated [6], presumably to protect them from degradation. The double stranded miRNA is then unwound, and the sense strand binds to an Argonaut protein, which is part of the RNA-induced silencing complex (RISC), triggering the specific destruction of RNA molecules that containing similar (4 or less mismatches) sequences to that of the miRNA [3]. Thus, miRNAs are endogenously expressed RNAs that may be expressed basally and accumulate. For example, in *Arabidopsis*, miR157 is expressed at a basal level in seedlings, leaves, stems, flowers and siliques (seed pods), but is most highly expressed in seedlings, whereas miR171 is most highly expressed in flowers. Alternatively, miR167 accumulates in all tissues except the stem. These observations reflect either differential transcription of the miRNA genes with different processed precursors or tissue-specific differences in the *Arabidopsis* miRNA processing machinery [2].

MiRNAs have been implicated in many areas of plant development [7]–[9] and responses to abiotic stresses [10]–[12]. Thousands of miRNAs have been identified across the plant kingdom, and to date, for example, there are 4,517 *Oryza sativa* miRNA sequences and 1,938 for *Arabidopsis thaliana* on miRNEST (version 2.0, 10 October 2013) [13]. This feature has allowed for cross-species comparison, and it is now clear that many plant miRNAs are strictly conserved across the plant kingdom [12], [14], while a smaller proportion appear to be species specific [15]. The evolutionary conservation of miRNA has allowed their identification in different genomes, including *Glycine max* [16], *Solanum tuberosum* [17] and *Malus domestica* [18]. In the papaya, 24 [19] and 75 [20] conserved miRNAs were identified via analyzes of small RNA deep sequencing data and the genomic sequence. Additionally, the high complementarity of plant (but not animal) miRNAs to their targets [21] allows for the prediction of their targets and thus their function within the plant [5].

Changes in miRNA expression are also associated with viral infection in *Arabidopsis* [22], and viral protein-induced alterations in miRNA expression have been associated with symptom development in *Nicotiana tabacum* [23]. A recent study [4] reported both increases and decreases in miRNA titres in tobacco following viral infection, and increases in specific miRNAs correlated with the degree of symptoms. It is theoretically possible that miRNAs could directly target viruses, as siRNAs display this capability, but this is not likely to be important in nature due to the ease by which viruses could avoid target specificity via mutation [24]. However, miRNAs do regulate plant resistance genes, such as the intracellular nucleotide binding (NB)-LRR immune receptors [25], making them valid candidates for indirect viral resistance.

**Figure 1:**
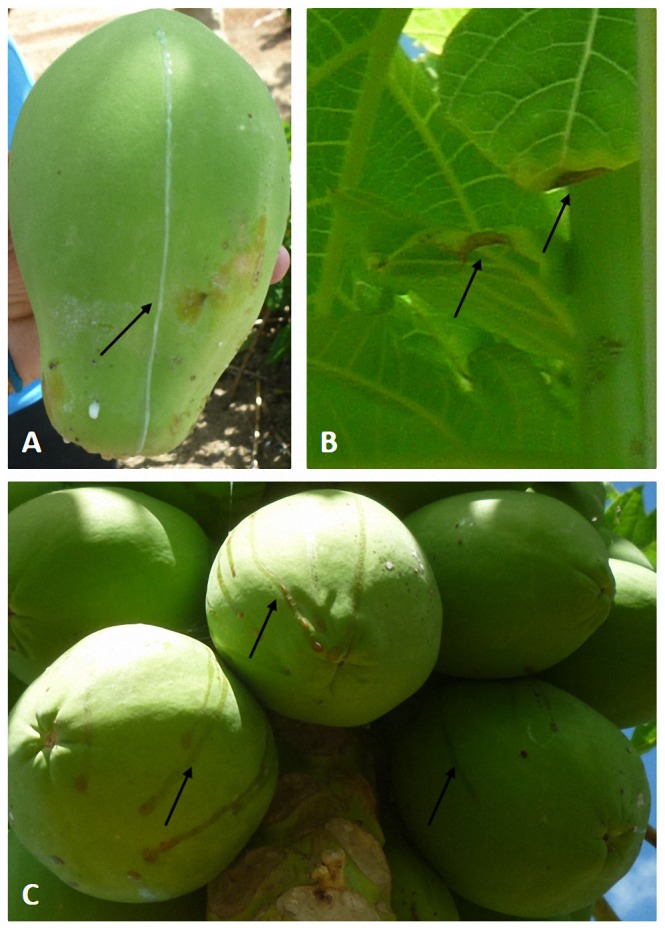
Symptoms of PMeV infection in *Carica papaya*. As opposed to most viruses, PMeV appears to reside primarily in lactifers, where it modifies potassium levels and osmotic balance, leading to rupture of cells and exudation of fluid and translucent latex from the fruits (A) and young leaves. The latex oxidises after atmospheric exposure, resulting in small necrotic lesions on the edges of young leaves (B) and a sticky latex on the fruits (C) that makes them unacceptable for consumption.

Papaya sticky disease, or “meleira”, is an important disease of the papaya (*Carica papaya* L.) that is capable of causing complete crop loss. Papaya tissues contain lactifers that maintain latex under high pressure so that it exudes upon injury as part of the defense mechanisms of the plant [26]. Tissues infected with papaya sticky disease spontaneously exude a translucent form of latex that is rapidly oxidised and darkens, rendering the fruit unsalable [27] (Figure 1A-C). The causal agent of sticky disease has been identified as *Papaya meleira virus* (PMeV), a 12 kbp dsRNA virus that presents as 50 nm spherical particles in infected tissues [28]. In contrast to most viruses, PMeV appears to reside primarily in lactifers where it modifies potassium levels and the osmotic balance, leading to rupture of cells and latex exudation [29].

So far, the factors involved in the onset of meleira symptoms are not known. Many infected plants show no disease symptoms and therefore they constitute an inoculum source in the papaya plantation. For that reason, efforts have been focused on the study of papaya-PMeV interaction. Previously published data have described the effects of PMeV on the laticifers regulatory network [30] and the systemic effects of PMeV on infected papaya leaves [31]. It has been proposed that the 20S proteasome subunit is involved in PMeV infection [31]. Several studies have reported the involvement of the ubiquitin/26S proteasome system (UPS) in the signalling and regulation of interactions between plants and pathogens [32]–[34], particularly viral exploitation and interference with the UPS [35]–[37].

Known plant microRNA sequences from the Plant miRNAs Database were used to search for potential microRNAs in papaya. Phylogenetic conservation analysis of the identified miRNAs was performed to assess the divergence between species. To understand the unique plant-virus interaction between PMeV and *C. papaya* and consequently the mechanisms involved in the onset of meleira symptoms, we investigated the miRNA response of the plant to infection, beginning with UPS. We studied four microRNAs that target components of 20S and 26S proteasomal degradation process and seven microRNAs that target components of other stress response pathways. The expression of each was compared using real-time PCR in healthy and infected papaya leaf tissue.

## Materials and Methods

### Prediction of miRNA hairpin and miRNA targets in *C. papaya* assembly

Mature miRNA sequences were downloaded from the Plant MicroRNA database (http://bioinformatics.cau.edu.cn/PMRD/). In total, 4,251 miRNA sequences were used to search forknown hairpin miRNA structures (miRNAs assigned with *akr* were removed from the analysis). The MIRcheck software package [38] was used to identify hairpins. The Patscan algorithm was used to identify target genomic sequence matches to known miRNAs, and it was set to run with 3 substitutions and no insertions/deletions (3 0 0). The genomic fragment of 350 flanking nucleotides to each side of the miRNA matching site was retrieved and used to predict secondary structures for each putative miRNA locus using the RNAfold programme in the Vienna RNA package (V 1.8.5, http://www.tbi.univie.ac.at/RNA/). The predicted secondary structure was then evaluated with parameters of known plant miRNA hairpins. Mature miRNA sequences were used to identify putative miRNA targets using the online version of psRNATarget (http://plantgrn.noble.org/psRNATarget/) [39]. In this investigation, we used the EST database (*C. papaya*, unigene, DFCI Gene Index (CAPAGI), version 1, released on 2010-05-27). The rules used for plant target prediction are based on two important analysis functions: i) reverse complementary matching between the miRNA and the target transcript using a proven scoring schema and ii) evaluation of target site accessibility by calculating the unpaired energy (UPE) required to “open” the secondary structure around the miRNA target site on mRNA. All putative targets regulated by miRNAs were subjected to gene ontology analysis. We extracted the unique IDs of targets and compared them with GO annotations of *C. papaya* TC annotations (available at http://compbio.dfci.harvard.edu/cgi-bin/tgi/tc_ann.pl?gudb=papaya).

Prediction of the folding structures of the identified precursors was carried out using Mfold (http://mfold.rna.albany.edu/?q=mfold/RNA-Folding-Form) [40]. The selected miR156 precursors were subjected to RNA sequence alignment using predicted secondary structures from R-Coffee (http://tcoffee.crg.cat/apps/tcoffee/do:rcoffee), and the resulting phylogenetic tree was verified.

### Choice of miRNAs for analysis

The 20S proteasome subunit is involved in PMeV papaya infection [31]. To assess the miRNA response to the infection, from the list of papaya miRNAs and their predicted targets, miR156, miR408, miR398 and miR162 were selected due to their involvement in proteasome degradation control. Seven other miRNAs were studied in papaya due to their involvement in the stress response, miR164, miR166, miR172, miR390, miR396, miR397 and miR399.The papaya miRNAs were confirmed as identical in sequence to those used in the commercially available TaqMan MicroRNA Assays (Life Technologies, Carlsbad, USA) for other plants, and therefore, these assays were used for analysis (Table S1).

### Plant material

A group of 40 plants of the same cultivar (*C. papaya* cv. Golden) were germinated and grown simultaneously in the field, at INCAPER experimental farm, Sooretama, Brazil. Young leaves samples from 6-month-old papaya plants were collected and immediately frozen in liquid nitrogen for transport to the laboratory. The samples were stored at −80°C until RNA isolation. The presence or absence of sticky disease symptoms was noted during collection.

### Assessment of viral load by real-time RT-PCR

The presence and viral load of PMeV was confirmed by real time RT-PCR as previously described [41]. Nucleic acids were extracted from papaya leaf samples (100 mg) using organic solvents and were precipitated using cold absolute ethanol and 3 M sodium acetate (pH 5.2) at −20°C overnight. Subsequently, nucleic acids were purified using a *mir*Vana™ miRNA Isolation Kit (Ambion, Austin, USA) to isolate total RNA, according to the manufacturer's instructions. The quality and quantity of the RNA was determined using a Nanodrop ND 1000 spectrophotometer. The presence of intact RNA was confirmed by RT-PCR of the actin gene. Nucleic acids were separated on 1% TBE agarose gels for 1.5 h at 80 V. After staining with ethidium bromide (15 ng ml^−1^), the gels were imaged using an L-HE-Pix/L-Pix IMAGE capture system (Loccus Biotecnologia, Cotia, Brazil).

RNA samples (600 ng) were mixed with 2 µL random hexamers (50 µM), 1 µL dNTP mix (10 mM) (Applied Biosystems, Carlsbad, USA) and DEPC water to a final volume of 12 µL. The samples were incubated at 96°C for 3 min, and cDNA was synthesised using a Super Script III kit (Invitrogen, Carlsbad, USA), following the manufacturer's instructions. Briefly, each sample received 4 µL SuperScript 5× buffer, 2 µL DTT (0.1 M), 0.1 µL RNaseOUT (40 U µL^−1^), 1.3 µL DEPC water and 0.6 µL 5× Super Script III (200 U µL^−1^) to achieve a final volume of 20 µL. The samples were incubated for 10 min at 25°C, 50 min at 50°C and 5 min at 85°C.

Real time RT-PCR was performed on a 7500 Fast Real-time PCR machine (Life Technologies, Carlsbad, USA) using PMeVreal primers [41]. Ten microliters of SYBR Green PCR Kit Master Mix (Applied Biosystems, Carlsbad, USA) and 6.5 µL of a solution of 2.8 mM of each primer diluted in 0.01 M Tris (pH 8.0) were added to the cDNA (3.5 µl). The mixture was incubated at 95°C for 10 min followed by 40 cycles at 95°C for 15 s and 60°C for 1 min. A melt curve of 95°C for 15 s, 60°C for 1 min and 95°C for 15 s was produced, and the data were analysed using the SDS Software System (7500 version 2.0.1, Applied Biosystems, Carlsbad, USA).

Relative abundance was estimated by the 2^−ΔΔCt^ method, after normalisation to cyclophilin. Cyclophilin (CYP), S-adenosyl methionine decarboxylase (SAMDC) and eukaryotic initiation factor 4A (EIF) are among the most stable genes when papaya is under biotic stress [42]. Each of these genes was evaluated as a possible reference gene using the geNorm algorithm [43] for papaya infected with PMeV. Following the comparison, cyclophilin was chosen for gene normalisation (Figure S1). Each sample was analysed in triplicate.

### Isolation of small and total RNA

Plant tissue (100 mg) was homogenised in liquid nitrogen, and either small (≤200 nt) or total RNAs (>200 nt) were isolated using the *mir*Vana™ miRNA Isolation Kit (Ambion, Austin, USA), following manufacturer's instructions. The small and total RNAs were eluted with 100 µl RNase-free water. The concentration, purity and integrity of the samples were determined by spectrophotometric measurement at 230, 260 and 280 nm (Nanodrop ND 1000 spectrophotometer, Nanodrop, Wilmington, USA), respectively. Only samples with a 260/280 ratio between 1.8 and 2.1 were used for further analysis.

### Reverse transcription of miRNA and total RNA

Reverse transcription was performed using a TaqMan MicroRNA Reverse Transcription kit (Life Technologies, Carlsbad, USA), following manufacturer's instructions. Briefly, 15 µl of reaction mixture (including 10 ng small RNAs (≤200 nt) or total RNA (>200 nt), 3 µl of TaqMan Small RNA Assay RT-primers or 3 µl of Random Primers (125 ng/µl), respectively) and 1 µl of Multiscribe reverse transcriptase were incubated at 16°C for 30 min, followed by incubations at 42°C for 30 min and 85°C for 5 min.

### Quantitative analysis of miRNA and target genes by real-time RT-PCR

Real-time PCR was performed on a 7500 Fast Real-time PCR machine (Life Technologies, Carlsbad, USA) using a TaqMan Small RNA Assay kit (Life Technologies) to analyse miRNAs and using a SYBR Green (Life Technologies) with a set of two PCR primers that flank the target region to analyse the target genes, according to the manufacturer's instructions. Each reaction consisted of 1.33 µl RT product, 10 µl of TaqMan Universal PCR Master Mix II (2X) or 10 µl of Sybr Green PCR Master Mix and 1 µl of primers (TaqMan Small RNA Assay) or 2 µl of forward and reverse primers at 10 µM, and the reactions were brought up to a final volume of 20 µl. The samples were incubated at 50°C for 20 s and 95°C for 10 min, which was followed by 40 cycles of 95°C for 15 s and 60°C for 60 s. The melting curve analysis for Sybr green reaction was assessed in samples incubated at 95°C for 15 s, 60°C for 60 s and 95°C for 15 s.

The amplification curve was plotted, and cyclophilin was used for normalisation [42]. Each miRNA and each target gene was analysed in duplicate. The relative expression of each miRNA or target gene in healthy and infected plants was calculated using the 2^−ΔΔCt^ method. The normalised miRNA or target gene level of the no detectable virus (control) sample was set as 1.0, and the samples were adjusted relative to this value. The results were analysed statistically by one-way analysis of variance (ANOVA) and Tukey's test (P<0.05).

## Results and Discussion

### In silico identification of conserved miRNAs

**Figure 2:**
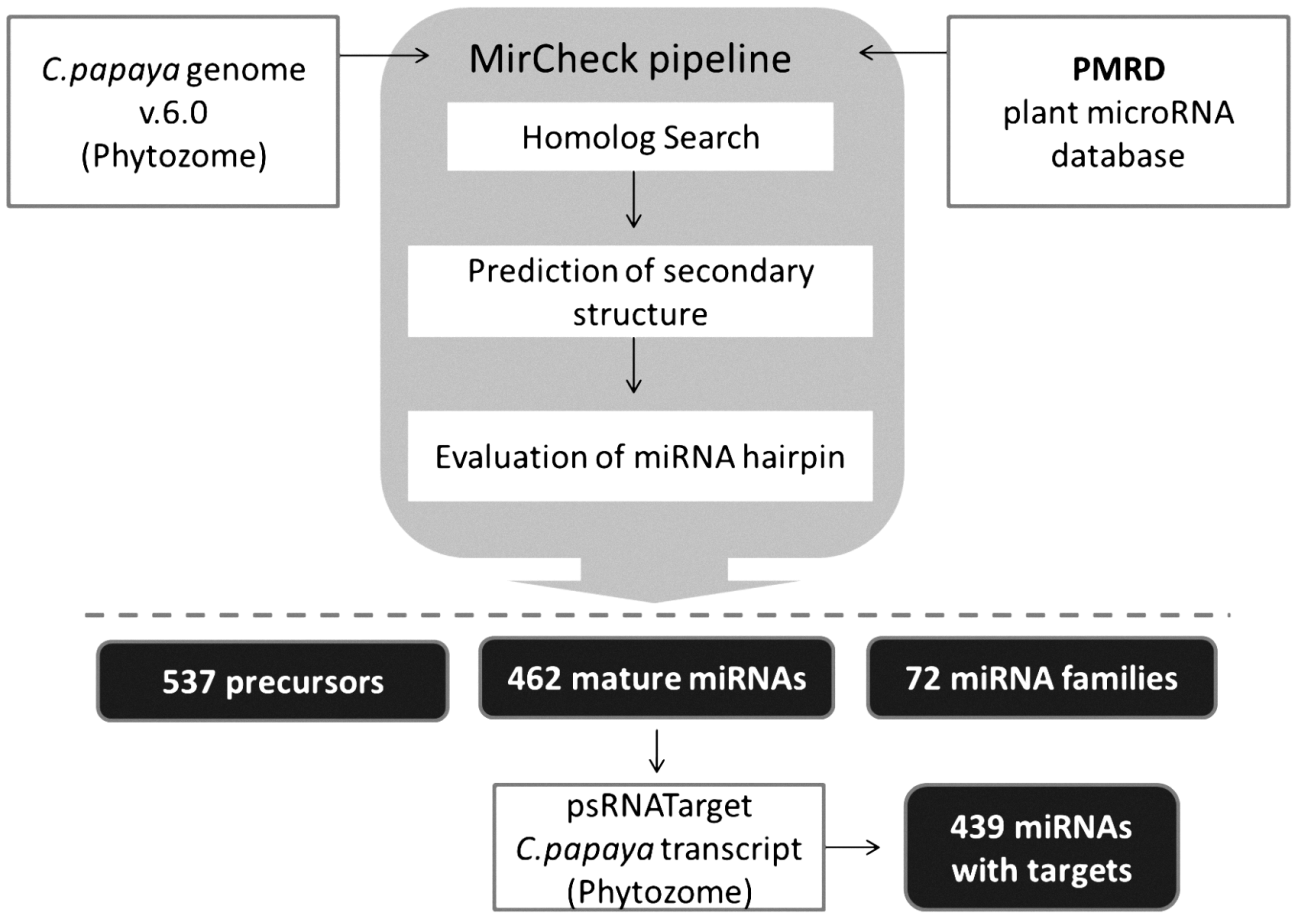
The pipeline of miRNAs search procedure and putative targets in the *C. papaya* genome. Flowchart of the principal steps for prediction of secondary structures of known miRNA and their putative targets in the *C. papaya* genome. Part of the flowchart represents the MirCheck pipeline.

**Figure 3:**
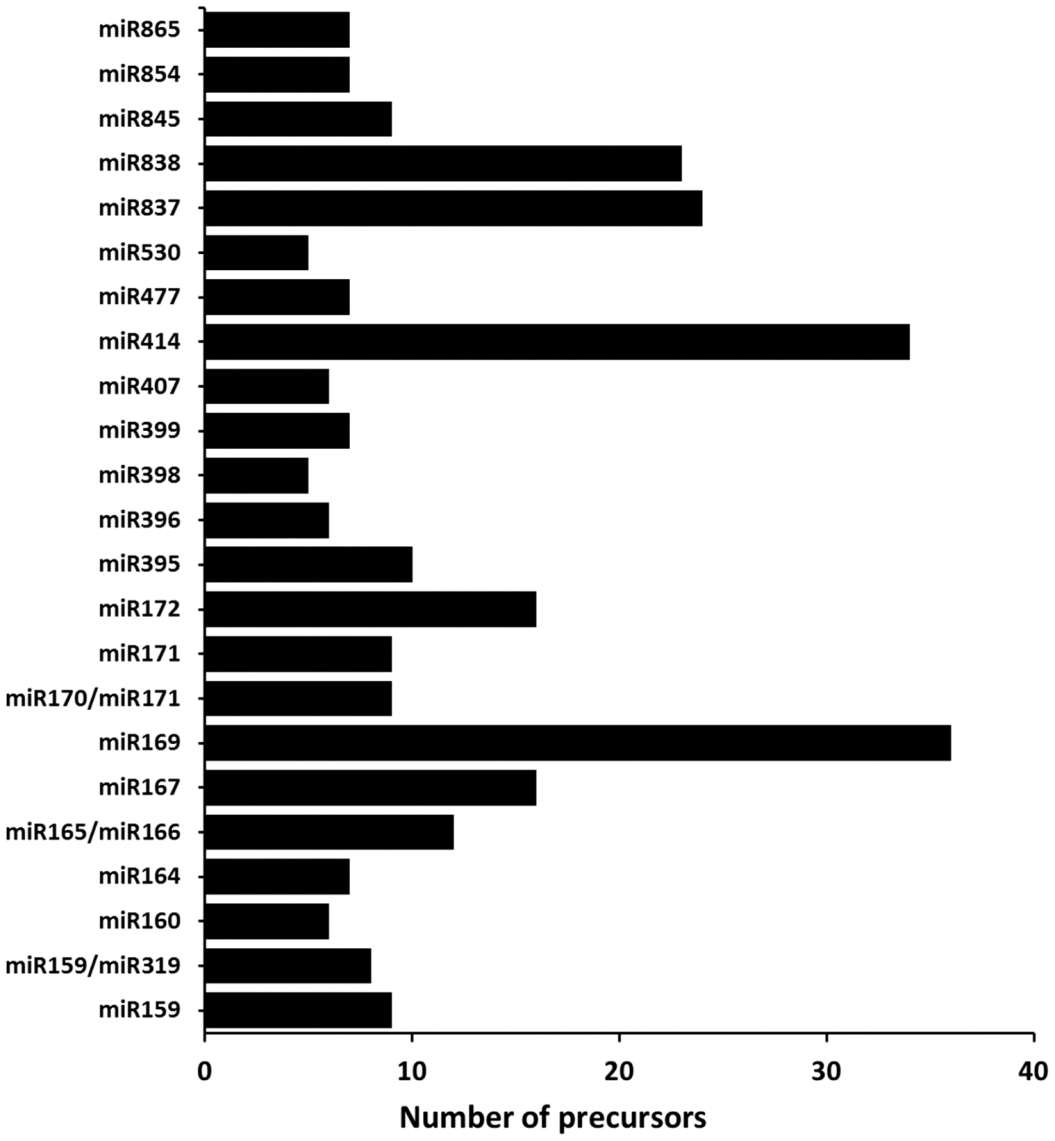
Analysis of *C. papaya* miRNA precursors. Number of precursors identified in each miRNA family (only miRNAs with more than 5 precursors are presented).

The conservation of mature miRNAs between plant species has enabled a computer-based approach to predict the secondary structures of the sequences surrounding miRNA. We used 4,251 plant mature miRNA sequences from the PMRD database to search for hairpin structures in the *C. papaya* genome. Known miRNAs were mapped to the *C. papaya* genome, and the flanking sequences surrounding miRNAs were retrieved. The hairpin structures were generated and evaluated using the MiRCheck pipeline and RNAfold. Figure 2 describes the pipeline and the main results of the miRNA search. Recently, 24 [19] and 75 [20] conserved miRNAs were identified in papaya. We detected 462 known miRNA sequences in 537 hairpins in the *C. papaya* genome; additionally, these miRNAs were classified into 72 miRNAs families. Of the 75 miRNAs sequenced by Liang *et al* [20], 66 were found in our *in silico* analysis, including of all miRNAs studied in this work. Table S2 displays the miRNA and precursors sequences that matched the criteria described in the MiRcheck pipeline. The length of miRNA precursors ranged from 55 to 371 nt, and the number of precursors per miRNA family varied, with some miRNA families, such as miRNA 169, having more than 30 members and other families, such as miRNA319, having only one (Figure 3). Only miRNAs with more than 5 precursors were represented. We analysed the distribution of miRNA sequences according to the first nucleotide of the 5′ end. Our results indicate that the majority of miRNAs had uridine (U) at the 5′ end (44%), corroborating the data described by Baumberger and Baulcombe [44], that showed a preferential association of the AGO1 protein with small RNAs containing 5′- terminal uridine. AGO1 is implicated in plant defense mechanisms, notably in plant antiviral immunity (reviewed in [45]). In contrast, viral proteins that target AGO1 inhibit silencing of the viral RNA by the host [45], [46].

### Phylogenetic conservation analysis of identified miRNAs

**Figure 4:**
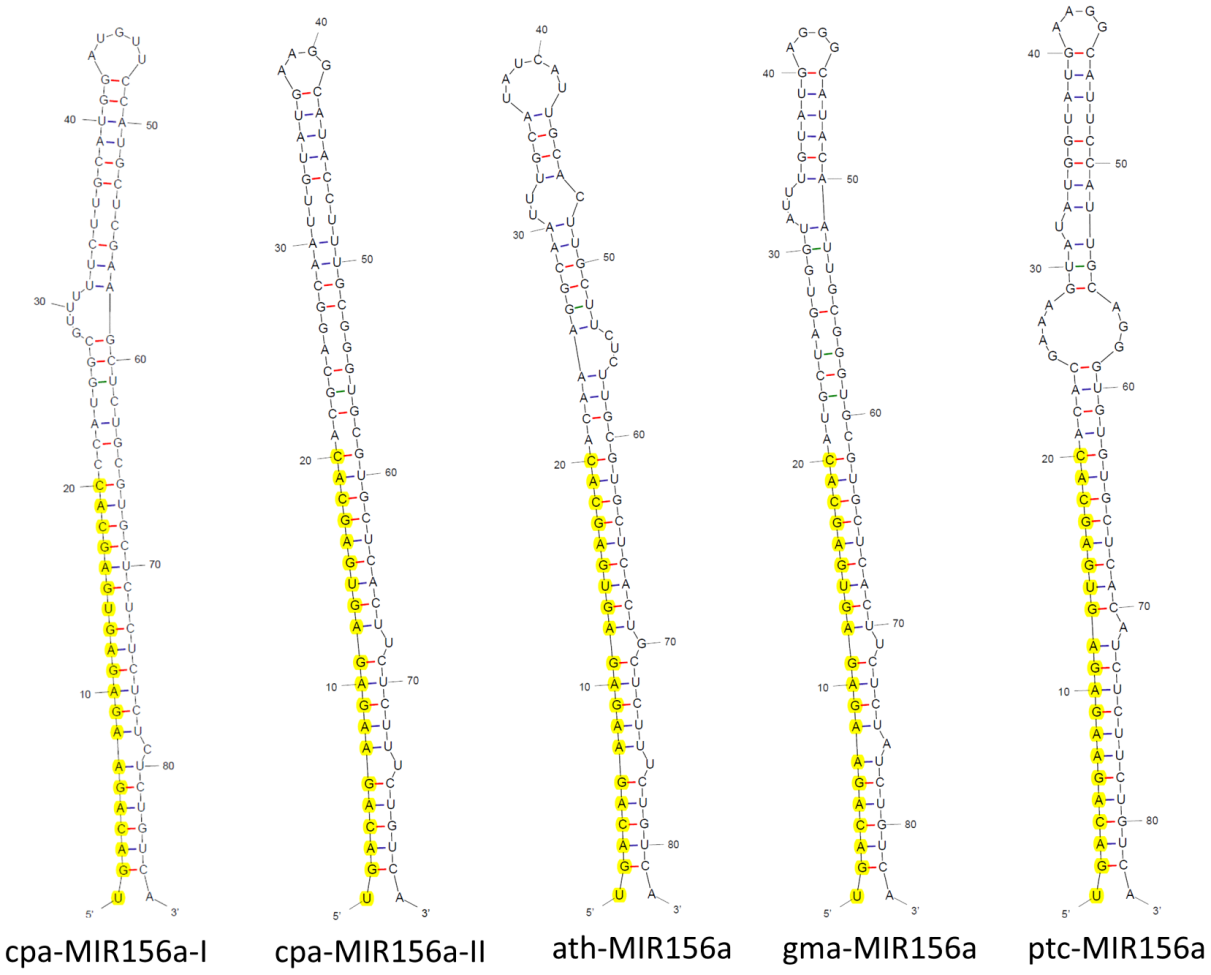
Secondary structures of selected MIR156a ortholog genes in *C. papaya*, Arabidopsis, soybean and Populus. The mature miRNA is highlighted (yellow) in stem-loop structures obtained using Mfold.

**Figure 5:**
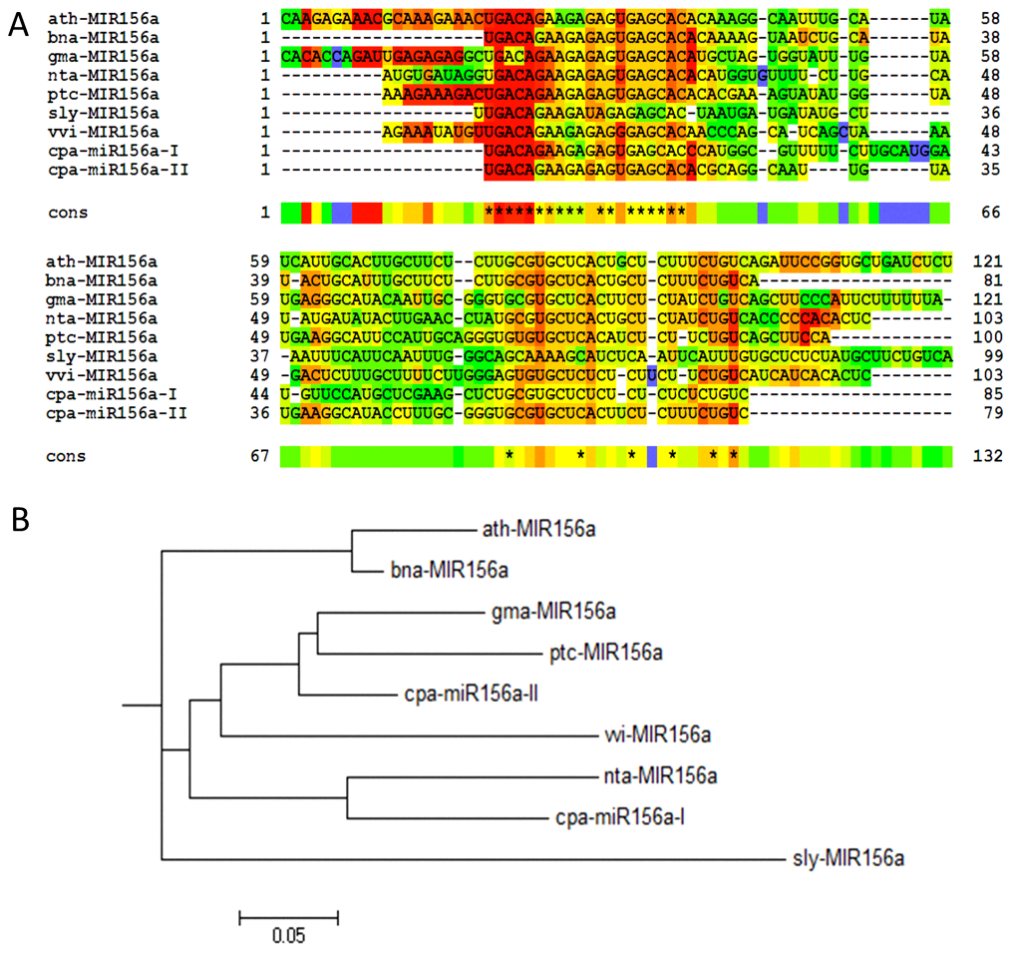
Phylogeny of *C. papaya* MIR156 orthologous precursors with dicot species. R-coffee alignment of *C. papaya* identified precursors (cpa-MIR156a-I and cpa-MIR156a-II) with MIR156a orthologs from *A. thaliana*, *B. napus*, *G. max*, *N. tabacum*, *P. trichocarpa*, *S. lycopersicum* and *V. vinifera*. Nucleotides marked in red and orange have better alignment (A); resulting relationship tree of R-coffee alignment (B).

The fast-evolving characteristic of miRNA precursor, as opposed to miRNA, sequences in plant genomes may reflect the phylogenetic divergence between species [47]. Phylogenetic conservation analysis of miRNAs between closely related species provides supporting evidence, and it has been used to annotate miRNAs in recently described plant genomes [48]. The distinguishing hairpin structure of the same miRNA in a plant species provides information regarding the evolution of plant genomes. Using Mfold, we compared the MIR156 homolog precursors found in C. papaya with other dicots (Arabidopsis thaliana, Glycine max and Populus trichocarpa) (Figure 4). The secondary structures of cpa-MIR156-II from the C. papaya precursor and gma-MIR156 from G. max showed the highest similarity, and the miRNA site on the ath-MIR156a (A. thaliana) precursor was also very similar to cpa-MIR156-II. To address the relationship between cpa-MIR156 homolog precursors and MIR156 precursors from dicot plants already deposited at miRBase, we used the R-coffee tool. This tool aligns the precursor secondary structure sequences to predict phylogeny. As expected, the greatest similarity between the different dicot precursors was observed at the miRNA/miRNA* position sites (Figure 5A, red and orange highlighted areas). Based on the hairpin sequences, cpa-MIR156-II had a close relationship with gma, ptc and wi-MIR156, the last of which is from Vitis vinifera, while cpa-MIR156-I was closely related to nta-MIR156 (Figure 5B). Interestingly, both C. papaya MIR156 homolog precursors were associated with the Solanum lycopersicum (sly-MIR156) branch of the phylogenetic tree, as opposed to the Arabidopsis and Brassica napus (bna-MIR156) branch, which are from the same order as C. papaya (the Brassicales). Therefore, phylogenetic conservation analysis of the identified miRNAs in papaya suggested that different miRNAs might evolve at different rates within the same species, and the same miRNA might evolve at different rates between species [49].

### Computational prediction of genes regulated by miRNAs

**Figure 6:**
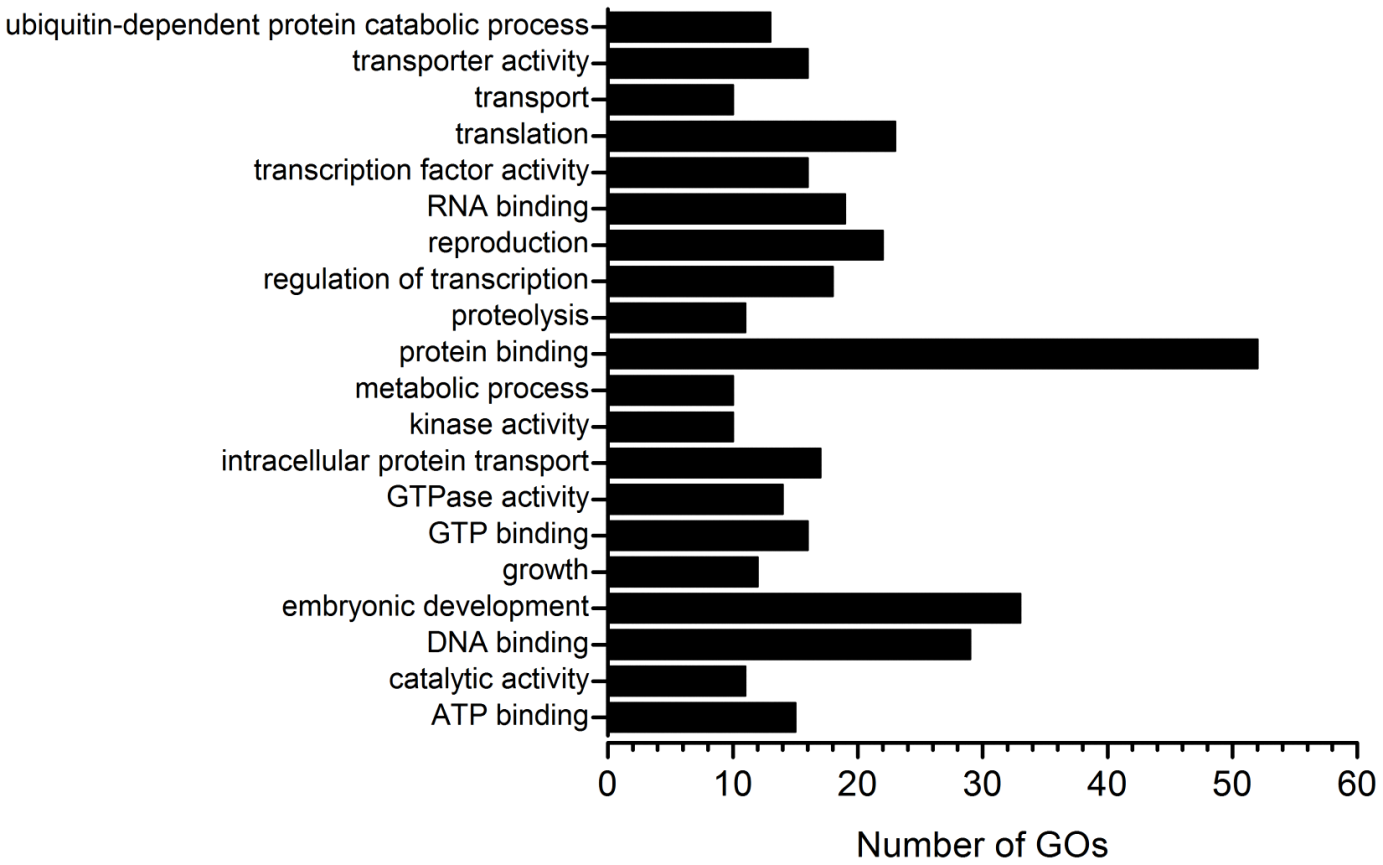
Gene Ontology enriched terms of putative miRNA targets. Bars indicate the number of GOs annotated as unique GO terms in *C. papaya* tentative consensus sequences.

The perfect, or near perfect, match between miRNAs and their targets in plants allows for the computational prediction of genes that can be regulated by miRNAs through cleavage, translation repression and, as more recently described, DNA methylation. We used the psRNATarget online tool to find putative miRNA-regulated targets at the *C. papaya* EST database. The results of this analysis are available in Table S3. We identified 3,525 putative targets in the entire EST dataset for 439 mature miRNAs sequences. Analysis of the *C. papaya* Tentative Consensus (TC) EST assembly identified 1,016 targets for 406 miRNA sequences, with an average of 2.5 targets *per* miRNA discovered. Next, we extracted the unique IDs of targets identified with “TC” to find overrepresented biological functions. The most representative GO term was protein binding, with 52 GO numbers (Figure 6). Many of the targets had GO terms involved in the regulation of transcriptional and metabolic process that are characteristic of miRNA-based regulation. miRNAs have the potential to regulate targets belonging to a number of gene families that have different biological functions.

The involvement of miRNAs in plant defense against viruses has been well described. For example, infection of *Nicotiana tabacum* with different plant viruses altered the accumulation of certain miRNAs, and they observed a correlation between symptom severity and the alteration of different miRNAs [23]. Some of the targets found in papaya are involved in the ubiquitin-dependent protein catabolic process. Increasing evidence indicates that plants utilise this process during their immune response to pathogen invasion, emphasising the role of this pathway during plant-pathogen interactions. Although proteasomal degradation serves as an effective barrier to help plants ward off pathogens in some cases, it is can be used by the pathogen to enhance the infection process as well. Viruses, influence the ubiquitin pathway to enhance their own replication (reviewed in [32], [33], [35], [36]). Because viruses themselves can manipulate the host miRNA system for their own advantage [30], we investigate miRNA expression at a number of viral loads that correspond to different stages of infection.

### Identification of miRNAs influenced by PMeV

**Figure 7:**
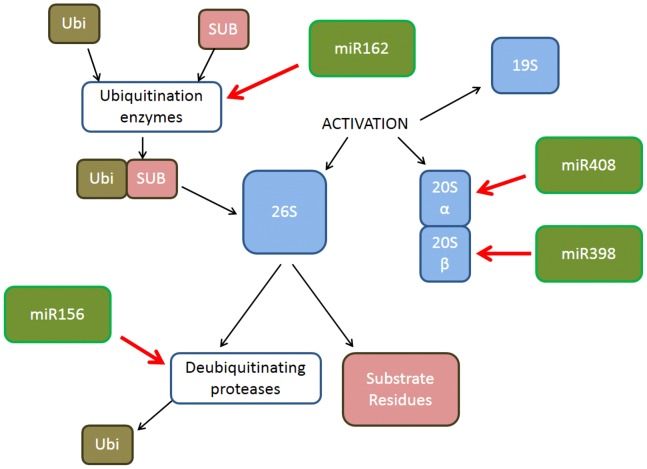
Relationship of miRNAs to proteasomal degradation. A schematic showing the relationship of miRNAs 156, 398, 408 and 162 to proteasomal degradation. The red arrows indicate the target genes of miRNAs. Proteasome-ubiquitin degradation involves the binding of ubiquitin (Ubi) to substrate (SUB). Polyubiquitinated proteins are recognised and degraded by the 26S proteasome, which consists of the 19S regulatory particle that recognises, selects and binds the polyubiquitinated proteins, cleaves the polyubiquitin chains and forwards the target polypeptide into the lumen of the 20S core particle, where proteolytic degradation takes place.

The UPS system plays an important role in plant vs. virus interactions. In papaya, the 20S proteasome subunit is upregulated during PMeV infection [31]. Thus, we decided to evaluate the expression of certain miRNAs whose targets participate in this pathway. Four miRNAs were identified as playing a role in the papaya proteasome in response to PMeV, miR156, miR162, miR398 and miR408. *In silico* analyses indicated that miR156 has 20 while the others have 21 nucleotides and that the miRNA targets were ubiquitin carrier protein and ubiquitin fusion-degradation protein like (miR156), polyubiquitin (miR162), proteasome subunit type beta (miR398) and proteasome subunit type alpha (miR408) (Table S3 and Figure 7). Seven other miRNAs, 164, 172, 390, 396, 397, 399 (21 nucleotides) and miRNA 166 (with 19 nucleotides), with target genes involved in important stress response pathways were studied in papaya infected by PMeV. *In silico* analyses indicated that the target of miR164, miR166, miR172, miR390, miR396, miR397 and miR399 were, among others, genes that encode for cystatin (CYS), NPK-1 related protein kinase (NPK1), hypersensitive-induced response protein (HIRP), GTP-binding protein (GBP), cytosolic ascorbate peroxidase (CAP), diphenol oxidase (DO) and extensin (EXT). The papaya miRNAs were confirmed as having an identical sequence to those of other plants used in the commercially available TaqMan MicroRNA Assays (Life Technologies, Carlsbad, USA), so they were used as supplied.

### Expression of miRNAs involved in proteasomal degradation and in the stress response

**Figure 8:**
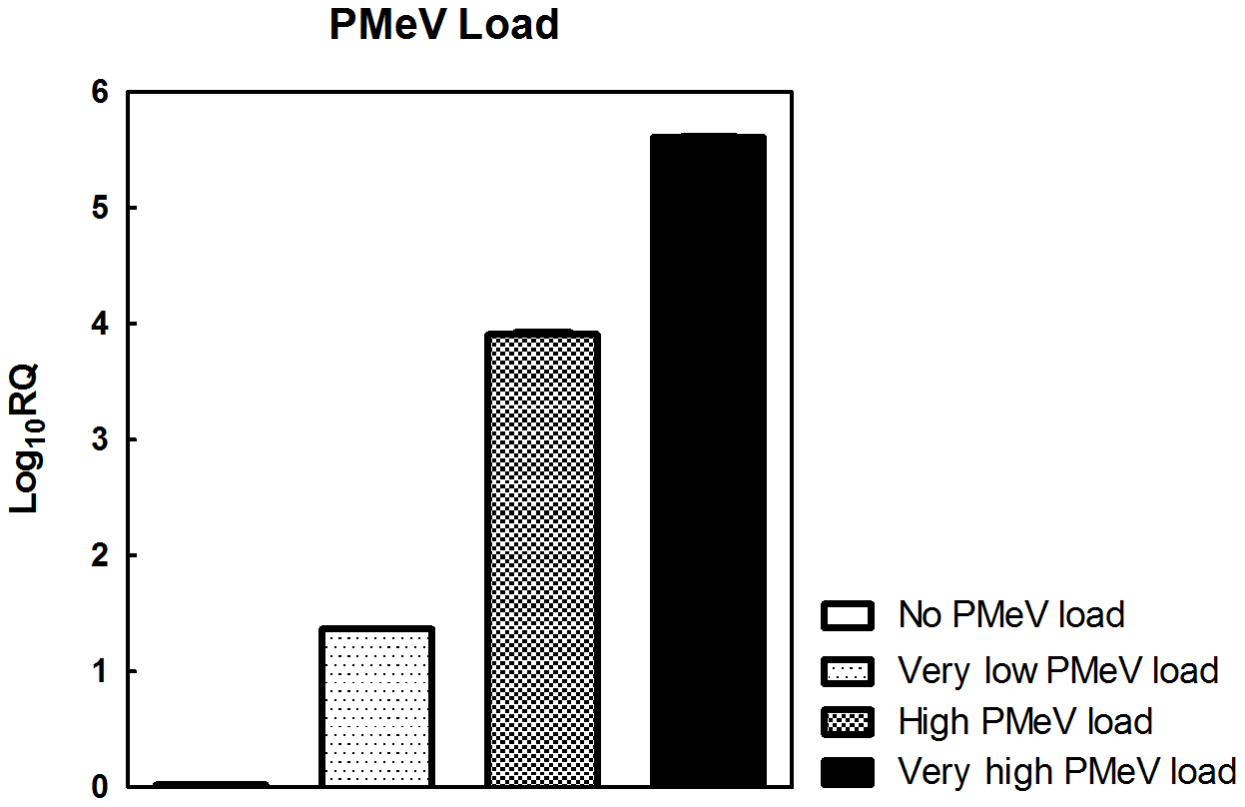
Relative quantification of PMeV load in *C. papaya* leaf samples. Relative quantification of PMeV following real time RT-PCR. The amplification curve was assessed, and cyclophilin was used for normalisation. The normalised level of No PMeV load (control) was set as 1.0, and the other PMeV loads were calculated in relation to the control. The following four samples were selected: 1 - No detectable virus, no symptoms; 2 - Low viral load (Log_10_RQ ∼1.4), no symptoms; 3 - High viral load (Log_10_RQ ∼4), with symptoms of sticky disease; and 4 - Very high viral load (Log_10_RQ ∼5.5), with symptoms of sticky disease.

After determining the relative abundance (Relative Quantification - RQ) of PMeV, the following samples were selected for miRNA analysis: 1 - No detectable virus, no symptoms; 2 - Low viral load (Log_10_RQ ∼1.4), no symptoms; 3 - High viral load (Log_10_RQ ∼4), with symptoms of sticky disease; and 4 - Very high viral load (Log_10_RQ ∼5.5), with symptoms of sticky disease (Figure 8).

**Figure 9:**
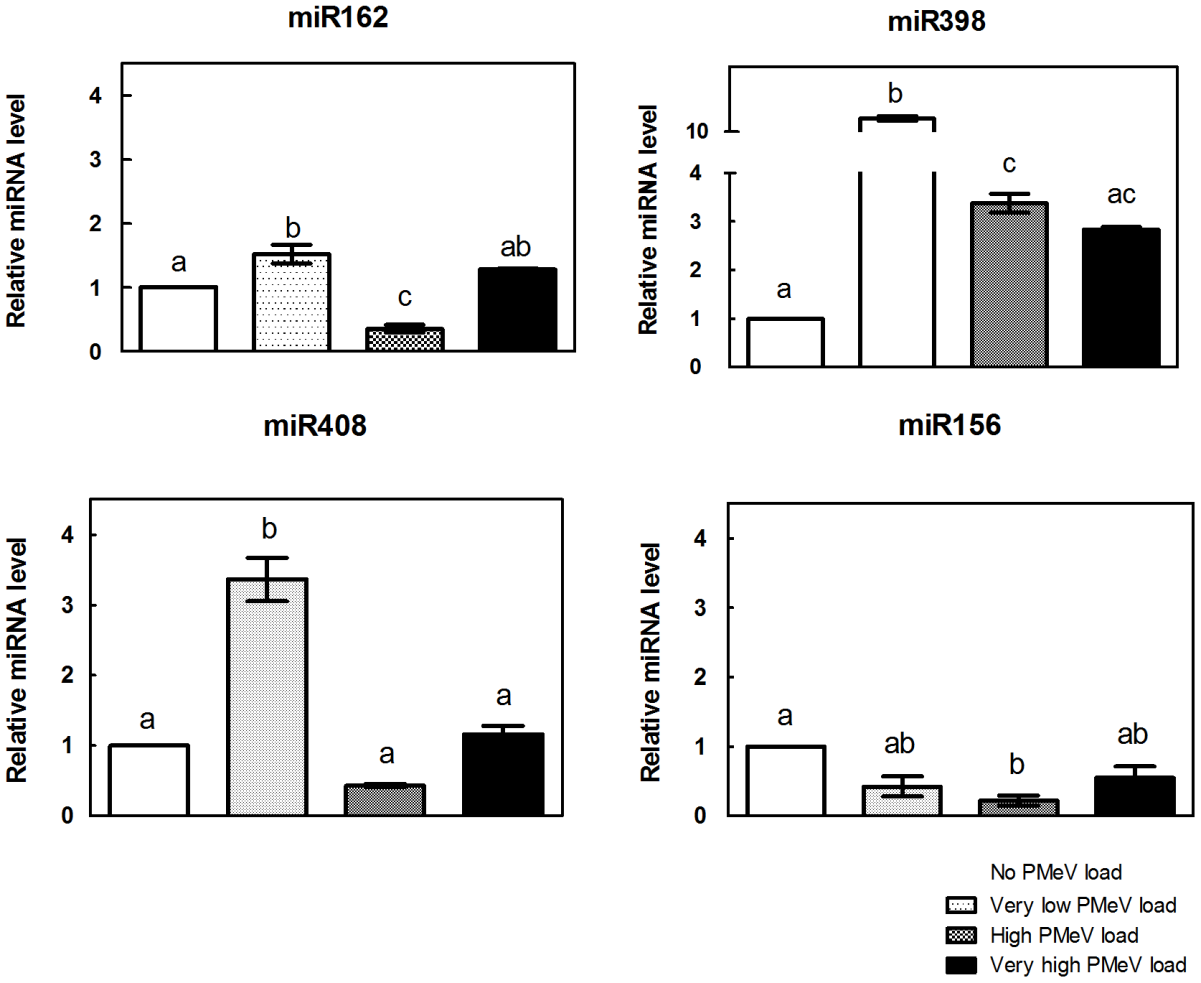
Relative quantification of miRNAs related to the UPS system in *C. papaya* leaf samples. MiRNAs were isolated from leaves, and the accumulation of a group of miRNAs associated with proteasome-ubiquitin degradation was assessed by RT-PCR using a TaqMan Small RNA Assay kit (Life Technologies). Comparison of the four samples: i) No PMeV load, ii) Very low PMeV load, iii) High PMeV load and iv) Very high PMeV load. The miRNA levels were normalized to cyclophilin (CYP) mRNA. The normalised miRNA level of No PMeV load (control) was set as 1.0, and the other PMeV loads were calculated in relation to the control.

**Figure 10:**
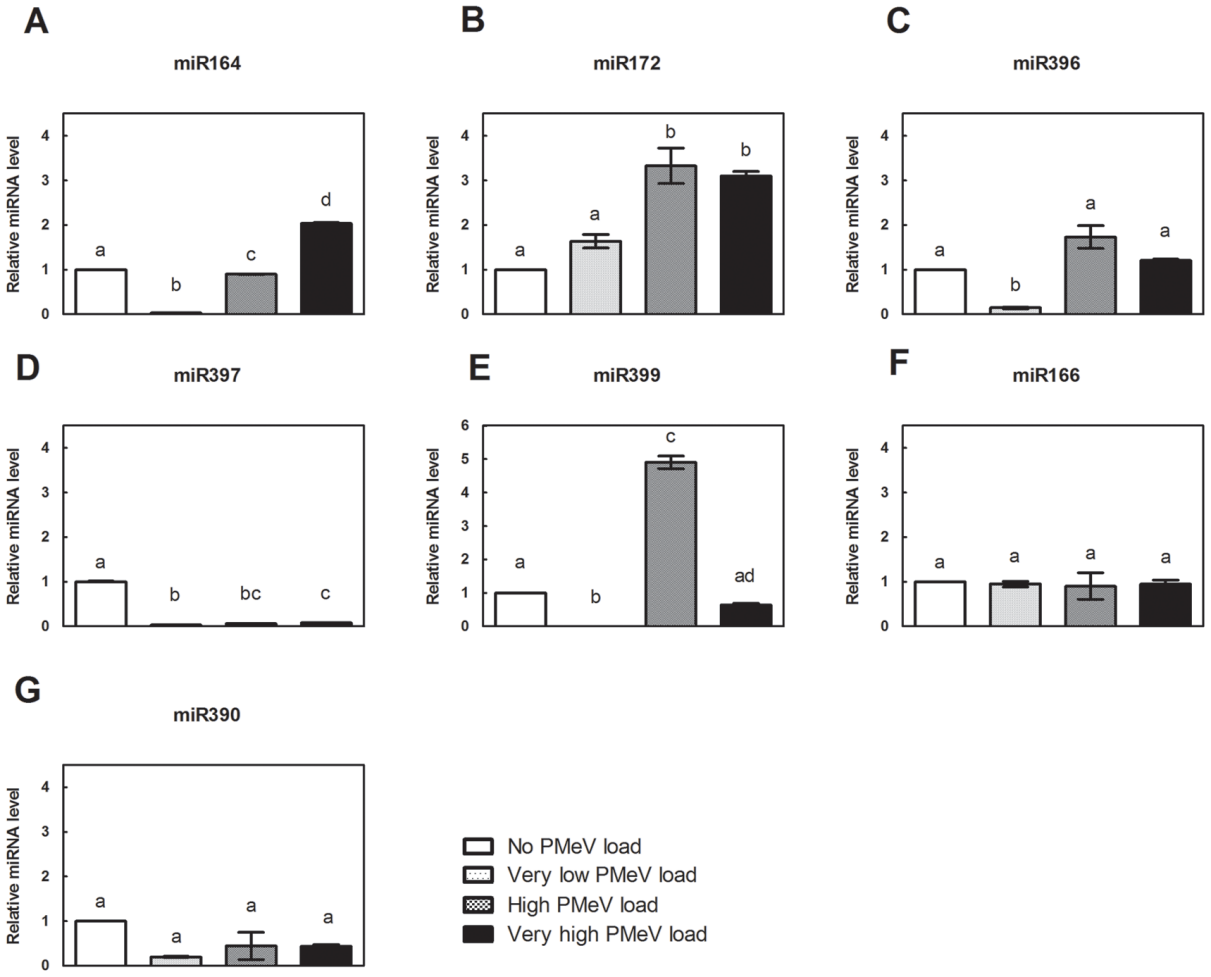
Relative quantification of miRNAs related to stress response pathways in *C. papaya* leaf samples. MiRNAs were isolated from leaves, and the accumulation of a group of miRNAs associated with stress response pathways was assessed by RT-PCR using a TaqMan Small RNA Assay kit (Life Technologies). Comparison of the four samples: i) No PMeV load, ii) Very low PMeV load, iii) High PMeV load and iv) Very high PMeV load. The miRNA levels were normalized to cyclophilin (CYP) mRNA. The normalised miRNA level of No PMeV load (control) was set as 1.0, and the other PMeV loads were calculated in relation to the control.

MiRNAs were isolated from papaya leaves, and the expression of a selection of miRNAs associated with proteasome-ubiquitin degradation and with stress response pathways was assessed by RT-PCR. Figure 9 shows the relative accumulation of miR156, miR398, miR162, and miR408 and Figure 10 of miR164, miR166, miR172, miR390, miR396, miR397 and miR399. MiRNA expression was normalised relative to the amount of miRNA species in the uninfected plants (group 1 – control group; set to 1.0).

The expression pattern in relation to PMeV load varied between the miRNAs. Three of the four miRNAs studied involved in proteasomal degradation, 162, 398 and 408, increased in response to an extremely low titre of PMeV (in an asymptomatic plant; group 2), most markedly miR398 (Figure 9B).

In the high viral load groups (in a symptomatic plant; group 3), the expression of miRNAs 162, 398 and 408 was considerably reduced (Figure 9A–C), and miR162 expression was significantly lower when no PMeV was detected (Figure 9A). Under very high viral load (in a symptomatic plant; group 4) the expression of miRNAs 162, 398 and 408 did not differ from the control group (Figure 9A–C). There was no significant difference between the high and very high viral load groups for miR398 and miR408 (Figure 9B–C), but miR162 expression was increased (Figures 9A).

The UPS system efficiently participates in the plant infection process as a defense mechanism against pathogens, including viruses (reviewed in [32], [33]). Basically, the cells need to remove unwanted proteins. These proteins are marked with ubiquitin polymer chains, recognised and become targets for proteolytic degradation via the proteasome (reviewed in [33], [35]). In this context, plants can inhibit the viral infection progress by degrading viral proteins that are important for infection. For example, the *Turnip yellow mosaic virus* (TYMV) replicase (RNA-dependent RNA polymerase) is targeted by the UPS system of infected plant cells, which affects viral replication efficiency [50].

Here, we show that PMeV modifies the transcription of several miRNAs involved in the modulation of genes related to the UPS system. There was a marked accumulation of miR162, miR398 and miR408 in healthy and asymptomatic plants with low PMeV load (group 2), whereas the accumulation of these miRNAs in plants with sticky disease symptoms (groups 3 and 4) was lower. The initial increase in the expression of miRNAs involved in the UPS system, likely reducing the UPS response, at first appears to be against the interest of the plant because viral proteins would no longer be targets of proteolytic degradation. However, previous reports indicate that suppression of the host ubiquitination machinery increases plant resistance to *Tobacco mosaic virus* (TMV) infection [51] and that the systemic movement of TMV and *Turnip mosaic virus* (TuMV) in *Nicotiana benthamiana* was prevented when the 26S proteasome was silenced using VIGS (Virus-induced gene silencing) [52]. Our results support this hypothesis, as despite infection, plants in group 2 did not manifest the disease, suggesting that the plant was somewhat resistant to PMeV. This phenomenon was clearer for miR398 (Figure 9B) presumably due to its proteasome subunit beta (target of miR398) carrier proteolytic activities [36]. Our findings correspond with those of Bazzini et al. [23], who reported a similar increase in miRNA expression in *Nicotiana tabacum* infected with various viruses.

In contrast, the UPS system may represent an opportunity for the pathogen to increase its infectivity. In some cases, viral proteins appear to impinge the UPS to induce host protein degradation or disrupt cellular signalling pathways; therefore, inhibiting plant defense mechanisms. In other circumstances, viral proteins themselves are targets of ubiquitination because their accumulation is detrimental to the host. Thus, removing excess viral proteins creates a favourable cellular environment and maintains host viability (reviewed in [32], [36], [37]). The expression of miR162 in plants corresponding to the onset of disease symptoms (group 3) was lower than in the control group, suggesting that the process of ubiquitination, which is necessary for UPS system maintenance, was upregulated. However, if PMeV proteins were targeted for ubiquitin-proteasome degradation in papaya, it was not sufficient to impair PMeV infection in these cases. One explanation for this observation is that the virus uses the UPS system for its own purposes, promoting the ubiquitination of excess viral proteins or host proteins during infection.

Viruses encode silencing suppressor proteins that impair the accumulation of host miRNAs [53]. Transgenic *Arabidopsis* expressing the suppressor protein from *Turnip mosaic virus* (TuMV), HCPro, demonstrate altered accumulation of miRNAs and presents symptoms that are similar to those of DCL1 mutant plants, suggesting that HCPro suppresses one or more of the steps downstream of DCL1-dependent processing [54]. The suppressor proteins may also interfere with miRNA-guided cleavage of target mRNAs by inhibiting the silencing of the target mRNA. For example, the *Beet yellows virus* p21 suppressor protein and the p19 of *Tomato bushy stunt virus*, interact with the miRNA, inhibiting their incorporation into active RISC and target cleavage [55].

Viral suppressor proteins are pathogenic factors, and therefore, they determine the onset of symptoms and disease development by interfering with the miRNA pathway of the host, which is necessary for the normal development of the plant [53], [56]. Thus, reducing miRNA expression and the appearance of sticky disease symptoms in plants with high viral load (groups 3 and 4) appears to be a consequence of the expression and accumulation of viral suppressor proteins.

To understand the systemic effects of PMeV in papaya, Rodrigues et al. [31] conducted a comprehensive proteomic analysis of leaf samples from healthy and diseased plants, and they found that 20S proteasome subunits are upregulated during infection. Additionally, the expression of miR398 and miR408, whose targets in papaya are 20S proteasome subunits, were reduced in response to increased PMeV load (groups 3 and 4), promoting the upregulation of genes encoding subunits of the 20S proteasome. These results corroborate with the work of Rodrigues et al. [31] and reinforce the idea that PMeV could take over the UPS system for its own benefit, increasing infectivity in papaya. This hypothesis seems even more likely because plants from groups 3 and 4 presented sticky disease symptoms.

The expression of miR162, miR398 and miR408 in plants with a very high viral load that were symptomatic for sticky disease (group 4) was similar to plants without PMeV, possibly because the virus had already gone through the process of replication and infection and was well established in the plant; thus, inhibition of the UPS was not necessary.

Interestingly, the response of miR156 was significantly different than the other three miRNAs studied here, as it was down regulated in response to PMeV infection rather than up regulated. Furthermore, there was no significant difference in expression between very low and very high titres (Figure 9D). It is possible that this miRNA is involved in deubiquitylation, a process that is potentially useful to the virus. For example, *in vivo* deubiquitylation of *Turnip yellow mosaic virus* RNA-dependent RNA polymerase protein leads to its stabilisation, which contributes to increased viral infectivity of plant cells [57].

In contrast, the expression of miRNAs involved in other stress response pathways reduced in an asymptomatic plant with very low PMeV load (group 2). There was a marked decreased of miRNAs 164, 396, 397 and 399, whereas miR172 did not differ from the control group (Figure 10A–E).

Figure 10 shows the relative accumulation of miR164, miR172 and miR399 in plants with sticky disease symptoms (groups 3 and 4), most markedly in miR399 that increased more than 5 times in plants with high viral load (group 3) (Figure 10A–B, E). When the viral RNA titre reached the highest level (group 4), the expression of miR396 and miR399 did not differ from the control group (Figure 10C, E). For miR397, however, the expression was significantly lower when no PMeV was detected (Figure 10D). The expression of miR166 and miR390 did not differ from the control group (Figure 10F–G).

**Figure 11:**
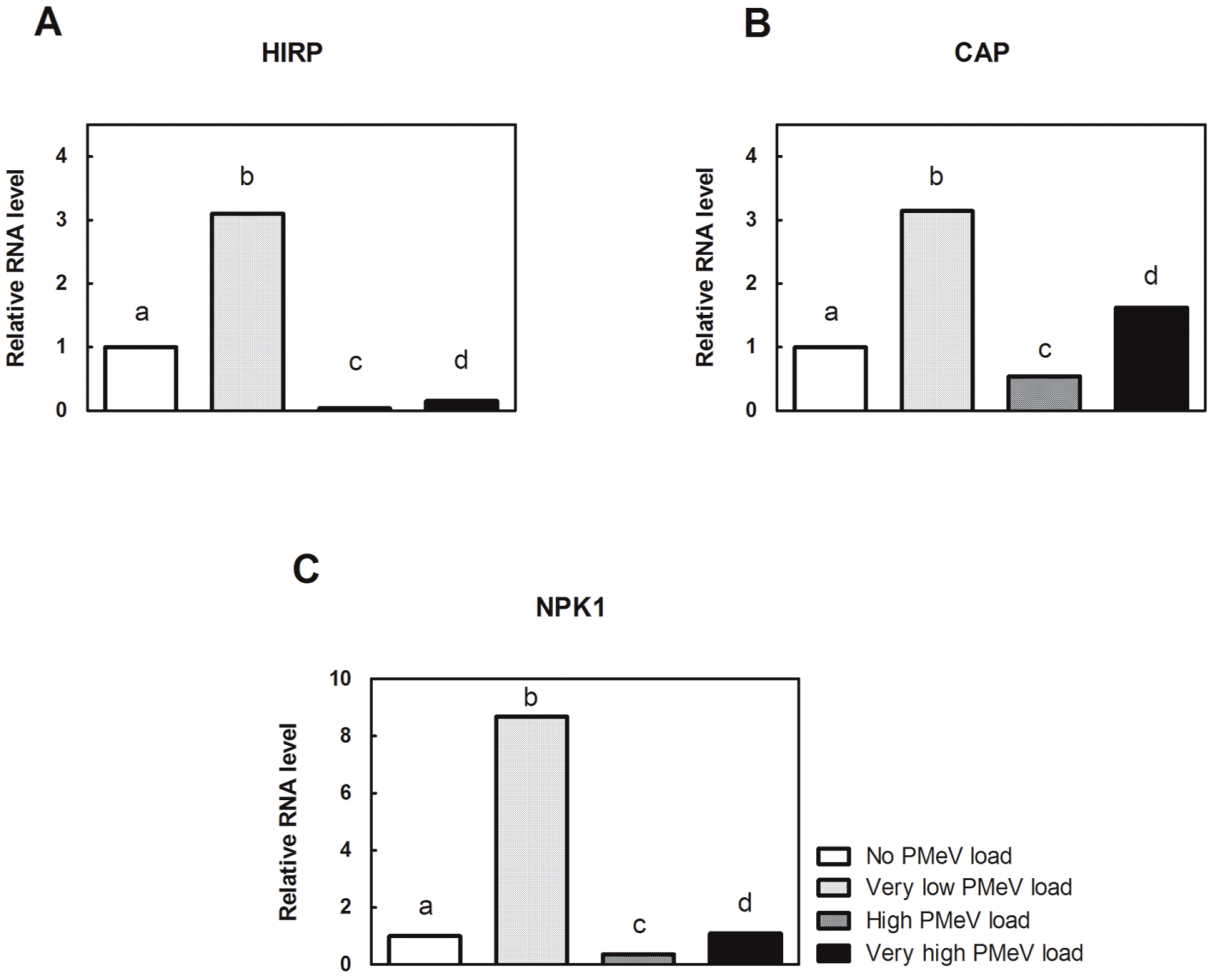
Relative quantification of RNAs in *C. papaya* leaf samples. mRNAs were isolated from leaves, and the accumulation of a group of RNAs associated with stress response pathways was assessed by RT-PCR using a SYBR Green (Life Technologies) with a set of two PCR primers that flank the target region to analyse target genes (Life Technologies). Comparison of the four samples: i) No PMeV load, ii) Very low PMeV load, iii) High PMeV load and iv) Very high PMeV load. The mRNA levels were normalized to cyclophilin (CYP) mRNA. The normalised mRNA level of No PMeV load (control) was set as 1.0, and the other PMeV loads were calculated in relation to the control.

Here, we show that PMeV modified the transcription of several miRNAs involved in stress response pathways. Interestingly, PMeV altered the expression of miRNAs that modulate genes involved in the reactive oxygen species (ROS) pathway. For example, there was a decrease of miR396 expression, whose targets are hypersensitive-induced response protein (HIRP) and cytosolic ascorbate peroxidase (CAP), in asymptomatic plant with very low viral load (group 2). Evidence suggests that ROS have a signalling function mediating defense gene activation and establishment of additional defenses, by redox control of transcription factors or by interaction with other signalling components like phosphorylation cascades [58], [59]. Our results support this idea since the expression of miR396 targets, as well as the miR166 target (NPK1-related protein kinase gene), increased in early infection (Figure 11A–C). Thus, the likely ROS production appears to be an attempt to control infection. Nevertheless, after the onset of sticky disease symptoms the expression of miR396 increased again decreasing the expression of genes related to ROS production and phosphorylation cascades pathways (Figure 11A–C). The expression of miRNAs involved in other stress response pathways, miR164, miR172 and miR399, followed the same profile of miR396 (Figure 10A–B, E).

A further increase in viral titre altered the expression of all miRNAs studied. Plant miRNAs negatively regulate mRNAs encoding important regulatory factors in plant development, stress response and pathogen defense mechanisms [7], [16]. Thus, altering the expression and function of miRNAs can cause abnormal plant development, resulting in disease symptoms [54]. The miRNAs used in this study are involved in the modulation of different genes implicated in plant developmental pathways. For example, miR162 targets the gene encoding Dicer-like1 (DCL1) in *Arabidopsis thaliana* [60]. DCL1 is a protein involved in the formation of miRNAs [61], [62], so miR162 also participates in the formation of miRNAs in plants. Alternatively, miR398 plays an important role in the response to abiotic stress, such as ozone and salinity, down-regulating the expression of Cu/Zn-superoxide dismutase [63]. Thus, the miRNAs analysed here are involved in different plant response pathways.

Thus, infection by PMeV altered the accumulation of miRNAs involved in the UPS system and also in stress response pathways of *Carica papaya*. The data presented here suggests that PMeV uses the plant UPS response as a strategy to favour its replication and maintain host viability and modifies the expression of miRNAs that modulate important defense genes, triggering the onset of sticky disease symptoms.

## Conclusions

The present work represents a comprehensive annotation of miRNAs and their targets in the papaya genome, and it will serve as a useful resource to complement the available molecular and genomic tools for the study of *C. papaya*. Eleven of the miRNAs identified were selected due to their association with the UPS system and with stress response pathways, and their expression was monitored during PMeV infection. The expression of these miRNAs changed, suggesting that PMeV can exploit and interfere with the UPS and with other stress response pathways. This was particularly noticeable through the expression of target genes involved in the ROS pathway, that play an important signalling role in plants controlling processes such as response to biotic stimuli, which changed during PMeV infection. Analysis of miRNAs appears to be useful for the study of PMeV infection and may help better understand and control this disease.

In addition, this study analysed the expression of miRNAs at different levels of viral load. Our study demonstrates that the plant response can vary depending on the viral titre and stage of infection and that a virus is capable of manipulating the host via the miRNA system.

## Supporting Information

Figure S1 **Stability of reference genes in PMeV infected papaya plants.** Three genes were tested as possible reference genes for papaya: cyclophilin (CYP), S-adenosyl methionine decarboxylase (SAMDC) and eukaryotic initiation factor 4A (EIF). Of these, the gene for cyclophilin was most stable in healthy and PMeV infected plants. The average expression stability of the remaining reference targets was estimated by geNorm.
(TIF)

Table S1 **Commercially available plant microRNA sequences used for analysis of papaya miRNAs.** Plant microRNA sequences were obtained from TaqMan MicroRNA Assays (Life Technologies). The table shows the name of the assay (as defined by Life Technologies), the sequence of interest and original organism associated with this sequence.
(XLSX)

Table S2 **MirCheck results of known miRNA precursors.** Overall information of precursors that matched the search criteria for the hairpin structure of known miRNAs in *C. papaya* assembled scaffolds.
(XLSX)

Table S3 **Putative targets of all miRNAs identified in** ***C. papaya*** **.** EST *C. papaya* data from Gene Index version 1.0 was used to search for potential new miRNA targets using psRNA Target.
(XLSX)
