# Supplementary figures and images for: Carica papaya MicroRNAs Are Responsive to Papaya meleira virus Infection
Source: PLoS One. 2014 Jul 29;9(7):e103401. doi: 10.1371/journal.pone.0103401 (PMC4114745; doi:10.1371/journal.pone.0103401)

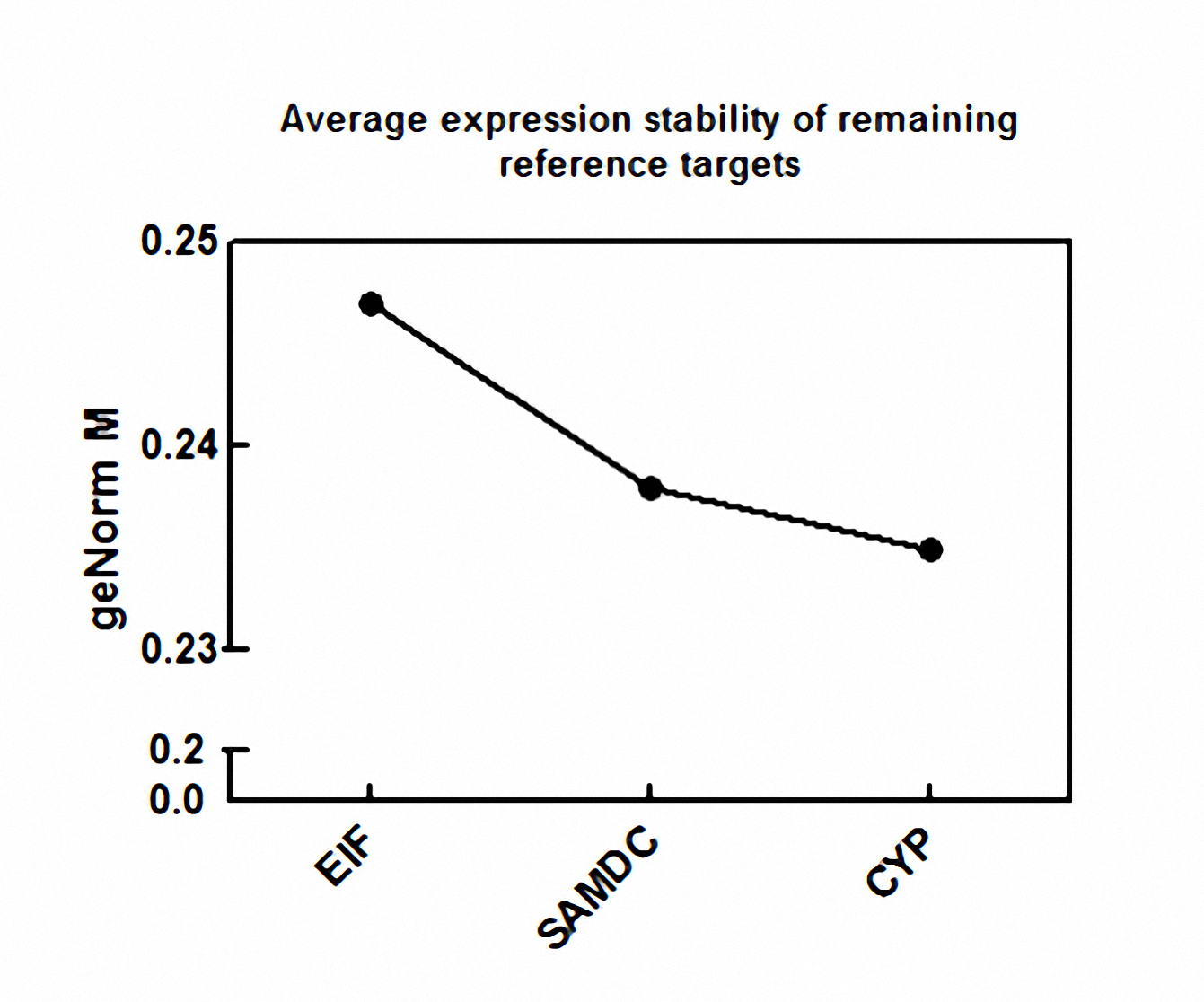

Supplement: Figure S1 — Stability of reference genes in PMeV infected papaya plants. Three genes were tested as possible reference genes for papaya: cyclophilin (CYP), S-adenosyl methionine decarboxylase (SAMDC) and eukaryotic initiation factor 4A (EIF). Of these, the gene for cyclophilin was most stable in healthy and PMeV infected plants. The average expression stability of the remaining reference targets was estimated by geNorm. (TIF) [file pone.0103401.s001.tif]
